# Establishment of immortalized ovarian stromal cell lines using Sendai virus vectors: a platform for studying tumor–stroma interactions and carcinogenesis

**DOI:** 10.1007/s13577-025-01324-6

**Published:** 2025-11-26

**Authors:** Masayo Okawa, Hiroaki Komatsu, Yasuhiro Kazuki, Kanako Kazuki, Genki Hichiwa, Kohei Hikino, Yuki Iida, Mayumi Sawada, Hiroyuki Kugoh, Shinya Sato, Mitsuo Oshimura, Tasuku Harada, Fuminori Taniguchi

**Affiliations:** 1 Department of Obstetrics and Gynecology, Tottori University School of Medicine, 36-1 Nishicho, Yonago, Tottori 683-8504 Japan; 2 Department of Chromosome Biomedical Engineering, Integrated Medical Sciences, Graduate School of Medical Sciences, Tottori University, Tottori, Japan; 3 Chromosome Engineering Research Center, Tottori University, Tottori, Japan; 4 Division of Genome and Cellular Function, Department of Molecular and Cellular Biology, Tottori, Japan; 5 Tottori University Hospital, Tottori, Japan

**Keywords:** Ovarian cancer, Borderline tumor, Immortalization, Stromal cell

## Abstract

We aimed to generate immortalized stromal cell lines from the ovarian and fallopian tube tissues of a single patient using Sendai virus (SeV) vectors and identify candidate stromal genes involved in ovarian carcinogenesis. Tissues were collected from a 48-year-old woman with endometrioid borderline tumors and endometriomas. Primary cultures were established from the right ovarian endometrioma, left ovarian surface, bilateral fallopian tube, and endometrial surface. Immortalization was achieved using SeV vectors encoding human telomerase reverse transcriptase (TERT), B lymphoma Mo-MLV insertion region 1 homolog (Bmi-1), and Simian virus 40 large T antigen (SV40T). Morphologically, the established cells exhibited spindle-shaped fibroblast-like features and expressed stromal markers (Vimentin-positive, Keratin-negative), confirming their stromal origin. Genetic and molecular changes associated with immortalization were evaluated via chromosomal analyses, transcriptome sequencing, and reverse transcription-polymerase chain reaction (RT-PCR). SeV-infected stromal cell lines retained their proliferative capacity for over 25 passages, whereas non-infected primary cells lost their epithelial characteristics and underwent senescence after five passages. Chromosomal abnormalities were more prevalent in stromal cells derived from the ovarian endometriomas, suggesting early genomic instability. Transcriptomic profiling and RT-PCR revealed upregulation of matrix metallopeptidase 1 (MMP1), pregnancy-associated plasma protein A (PAPPA), and C-X-C motif chemokine ligand 1 in cyst-derived stromal cells compared to those from the normal ovary and fallopian tube, implicating these genes in extracellular matrix remodeling and tumor–stroma crosstalk. We established immortalized ovarian and fallopian tube stromal cell lines using SeV-based vectors. The cyst-derived stromal cells exhibited early chromosomal instability and overexpression of MMP1 and PAPPA, supporting their potential role in ovarian carcinogenesis. These immortalized stromal cell lines provide a novel and stable platform for mechanistic studies and may contribute to biomarker discovery and therapeutic target development in ovarian cancer.

**Supplementary Information:**

The online version contains supplementary material available at 10.1007/s13577-025-01324-6.

## Introduction

Ovarian cancer remains the most lethal gynecologic malignancy, with late-stage diagnosis, recurrence, and chemoresistance driven in part by the tumor microenvironment. Among stromal components, cancer‑associated fibroblasts (CAFs) actively regulate invasion, angiogenesis, extracellular matrix (ECM) remodeling, stemness, and therapy resistance through paracrine signaling and direct cell–cell interactions [1]. Recent studies highlight specific CAF-derived factors that promote ovarian cancer progression, including non-canonical WNT5A signaling that sustains cancer stemness via the ROR2/PKC/CREB1 pathway [2], FGF7 (keratinocyte growth factor) that signals through FGFR2 to stabilize HIF‑1α and enhance proliferation, migration, and invasion [3], and stromal versican that cooperates with hyaluronan/CD44 to drive motility and peritoneal dissemination [4].

Despite their central importance, mechanistic studies of ovarian stromal biology have been constrained by the short replicative lifespan and heterogeneity of primary fibroblast/CAF cultures. The establishment of immortalized stromal cell lines—exemplified by hTERT‑transduced normal ovarian fibroblasts (NOF151‑hTERT)—has enabled reproducible dissection of TGF‑β/versican and other stromal pathways in ovarian cancer models [1]. Moreover, integrating immortalized stromal cells into advanced platforms (3D co‑culture organoids and microfluidic tumor‑on‑a‑chip systems) better recapitulates key tumor–stroma interactions relevant to drug response and resistance [5, 6].

Here, using non-integrating Sendai virus (SeV) vectors encoding hTERT, BMI1, and SV40T [7], we established immortalized ovarian stromal cell lines from tissues obtained from a single patient. Previous studies have demonstrated the successful use of SeV-mediated immortalization in mesenchymal stromal cells, leading to both stable cell line establishment and therapeutic applications. Building on these precedents, our approach highlights not only the feasibility of establishing ovarian stromal cell lines but also their potential translational utility—from disease modeling to therapeutic development—within the ovarian research field. We show that cyst‑derived stromal cells exhibit early genomic instability and upregulation of ECM/paracrine genes (MMP1, PAPPA, CXCL chemokines), positioning these lines as a stable, renewable platform to interrogate stromal contributions to ovarian carcinogenesis and to develop stroma‑targeted strategies.

## Materials and methods

### Patient background

A 48-year-old woman underwent a total hysterectomy and bilateral salpingo-oophorectomy for borderline right ovarian endometrioid tumor and endometriomas. The presence of endometriomas and endometrioid borderline tumors was confirmed by hematoxylin and eosin staining and immunohistochemical staining using paraffin block embedding. Simultaneously, endometrial, ovarian, and fallopian tube cancers were not observed histopathologically.

### Establishment of primary cell line

The entire tissue removed during the surgery was thoroughly washed with saline under aseptic conditions. The endometrium lining of the right ovary, surface of the left ovary, bilateral fimbriae of the fallopian tubes, and endometrial surface were cut into small pieces in 1 × phosphate-buffered saline (PBS) using a scalpel or ophthalmic forceps.

A 1% collagenase solution was prepared by dissolving lyophilized collagenase IV powder in Dulbecco’s modified Eagle’s medium (DMEM)/Ham’s F-12 medium with L-glutamine and sodium pyruvate (containing L-glutamine, pyruvate, and HEPES, but without phenol red) (DMEM/Ham’s F-12) (nacalai tesque, Cat No. 05177–15). Next, 5 mL of the 1% collagenase solution was dispensed into a 15-mL tube. Then the cells collected from the surgical specimen were mixed with this solution. The mixture was shaken at 37 °C at 100 rpm for 12–24 h using a small cooling shaker.

After shaking, the mixture was filtered twice through a 70-μm cell strainer (Corning, Cat No. 352350) into a 50-mL tube. The filtrate was centrifuged at 1300 rpm for 8 min at room temperature. After aspirating the supernatant, the cell pellet was re-suspended in 2 mL of DMEM/Ham’s F-12, followed by the addition of 8 mL of DMEM/Ham’s F-12 to achieve a total suspension volume of 10 mL. Then the suspension was filtered into a 50-mL tube using a 40-μm cell strainer (Corning, Cat No. 352340). The final filtrate was resuspended in DMEM/Ham’s F-12 supplemented with 1% penicillin–streptomycin solution (P-S) and seeded for culture in a CO₂ incubator at 37 °C.

The next day, the cells were gently washed with 1 × PBS(-) to remove blood cells and then cultured in DMEM/Ham’s F-12 supplemented with 20% fetal bovine serum (FBS) and 1% P-S. Once the cell population reached an appropriate density, the cells were seeded at a density of 2.0 × 10^5^ cells/well in a 24-well plate for SeV infection. The following day, the cells were infected with an SeV vector carrying three immortalization genes (human telomerase reverse transcriptase (hTERT), B lymphoma Mo-MLV insertion region 1 homolog, and Simian virus 40 large T antigen [SV40T]). The hTERT maintains telomere length, BMI1 suppresses the p16/p14 senescence pathway, and SV40T inactivates p53 and Rb, respectively, thereby providing complementary mechanisms for stable immortalization.

SeV vectors used for gene delivery and vector production were provided by ID Pharma, Inc.

SeV-infected (immortalized cell lines) and SeV-uninfected (parental cells) cells were passaged and expanded in 10-cm dishes. The medium was changed every 2–3 days with DMEM/Ham’s F-12 supplemented with 20% FBS and 1% P-S. Then the cells were sub-cultured at a 1:4 ratio in 10-cm dishes.

### SeV vector infection

The cells were seeded at a density of 2.0 × 10^5^ cells/well in a 24-well plate. The next day, cells were infected with SeV-hTERT (co-transduced with enhanced green fluorescent protein), SeV-Bmi1 (co-transduced with orange fluorescent protein [OFP]), and SeV-SV40T in a biosafety cabinet. The viral infection was performed following previously described protocols using a non-integrative and conditionally removable Sendai virus vector [7]. Then the infected cells were cultured in a 35 °C CO₂ incubator.

### Chromosome analysis

Each cell line was seeded in a 6-cm dish with 5 mL of DMEM/Ham’s F-12 supplemented with 20% FBS and 1% P-S. The medium was changed on the day before Carnoy’s fixation. When the cells reached 80–90% confluence, they were fixed as follows:

The cells were synchronized using Metaphase Arresting Solution (Genial Genetic Solutions) and Chromosome Resolution Additive and then treated with 0.075 M potassium chloride before fixation in Carnoy’s fixative.

The fixed cells were dropped onto a slide and stained with a QH staining solution, which included quinacrine (quinacrine mustard dihydrochloride; Sigma-Aldrich, Q2876) and Hoechst (bisbenzimide H 33258; Sigma-Aldrich, B2883). Multicolor fluorescence in situ hybridization (mFISH) was performed using a 24XCyte probe set (MetaSystems Inc.).

Metaphase images were captured using an Axio Imager Z2 fluorescence microscope (Carl Zeiss GmbH, Jena, Germany). Metaphase karyotypes were analyzed using Ikaros and ISIS software programs (MetaSystems).

### Human transcriptome base sequence analysis

Total RNA was extracted using an RNeasy Mini Kit (Qiagen, Cat. No. 74104, 74,106). RNA quality was assessed using the Agilent 2200 TapeStation, confirming that the RNA yield was ≥ 10.0 μg. Sequencing analysis was performed using the NovaSeq 6000 system (Illumina).

The sample was prepared using the TruSeq Stranded mRNA Library Prep Kit to generate libraries with fragment lengths of 354–368 bp. Sequencing was performed using the multiplex method with a read length of 101 bp.

Cluster analysis was performed using the k-means method, which classified the clusters into five categories based on the slope of the expression variation ratio. PCR primers corresponding to genes with more than a twofold change in expression were selected by comparing each cell line.

### Real-time PCR

Total RNA was extracted from each cell line using an RNeasy Mini Kit (Qiagen, Cat. No. 74104, 74,106).

DNase treatment was performed using a deoxyribonuclease (RT Grade) (NIPPON GENE, Cat. No. 313–03161) in combination with RNaseOUT™ Recombinant Ribonuclease Inhibitor (Invitrogen, Cat. No. 10777019).

cDNA was synthesized using a High-Capacity cDNA Reverse Transcription Kit (Applied Biosystems™, Cat. No. 4368814). Real-time polymerase chain reaction (RT-PCR) was conducted using the StepOnePlus™ Real-Time PCR System (Applied Biosystems) and TaqMan™ Fast Advanced Master Mix (Applied Biosystems™, Cat. No. 4444556) according to the manufacturer’s protocol. Gene expression levels were normalized to β-actin.

The PCR conditions were as follows: uracil-N-glycosylase incubation at 50 °C for 2 min, polymerase activation at 95 °C for 20 s, followed by 40 cycles of denaturation at 95 °C for 1 s and annealing/extension at 60 °C for 20 s.

### Immunocytochemistry

Cells cultured on chamber slides were fixed with 4% paraformaldehyde (PFA) for 15 min at room temperature. After fixation, the cells were neutralized with 0.1 M glycine in PBS for 15 min at room temperature. The cells were washed three times with PBS. Blocking solution consisted of 1% goat serum, 0.1% Triton X-100, and Protein Block Serum Free (Agilent Dako, Cat. No. X0909). The cells were then incubated with a blocking A solution for 15 min at room temperature. Subsequently, the cells were incubated with primary antibodies for 60–90 min at room temperature. Primary antibodies were applied as follows: Mouse anti-Vimentin monoclonal antibody (clone VIM 3B4; Progen Biotechnik, Cat. No. 65013, IgG2a) and Mouse anti-Keratin K5/K8 (pan-epithelial) monoclonal antibody (clone C22; Progen Biotechnik, Cat. No. 65031, IgG1). After three washes with PBS containing 0.1% Triton X-100 (PBST), cells were incubated with species-appropriate fluorophore-conjugated secondary antibodies (Alexa Fluor series, Invitrogen; diluted 1:500) for 1 h at room temperature in the dark. Nuclei were counterstained with TO-PRO-3 iodide (Thermo Fisher Scientific, Cat. No. T3605; 1:1000) for 15 min. Slides were washed with PBS, and chamber walls were removed before coverslips were mounted using ProLong™ Diamond Antifade Mountant without DAPI (Thermo Fisher Scientific, Cat. No. P36970). The mounted slides were stored at 4 °C overnight in the dark before sealing with nail polish. Images were acquired using a confocal fluorescence microscope (LSM series, Carl Zeiss) under identical exposure settings.

## Results

### Cell morphological characteristics and proliferative ability

**Fig. 1:**
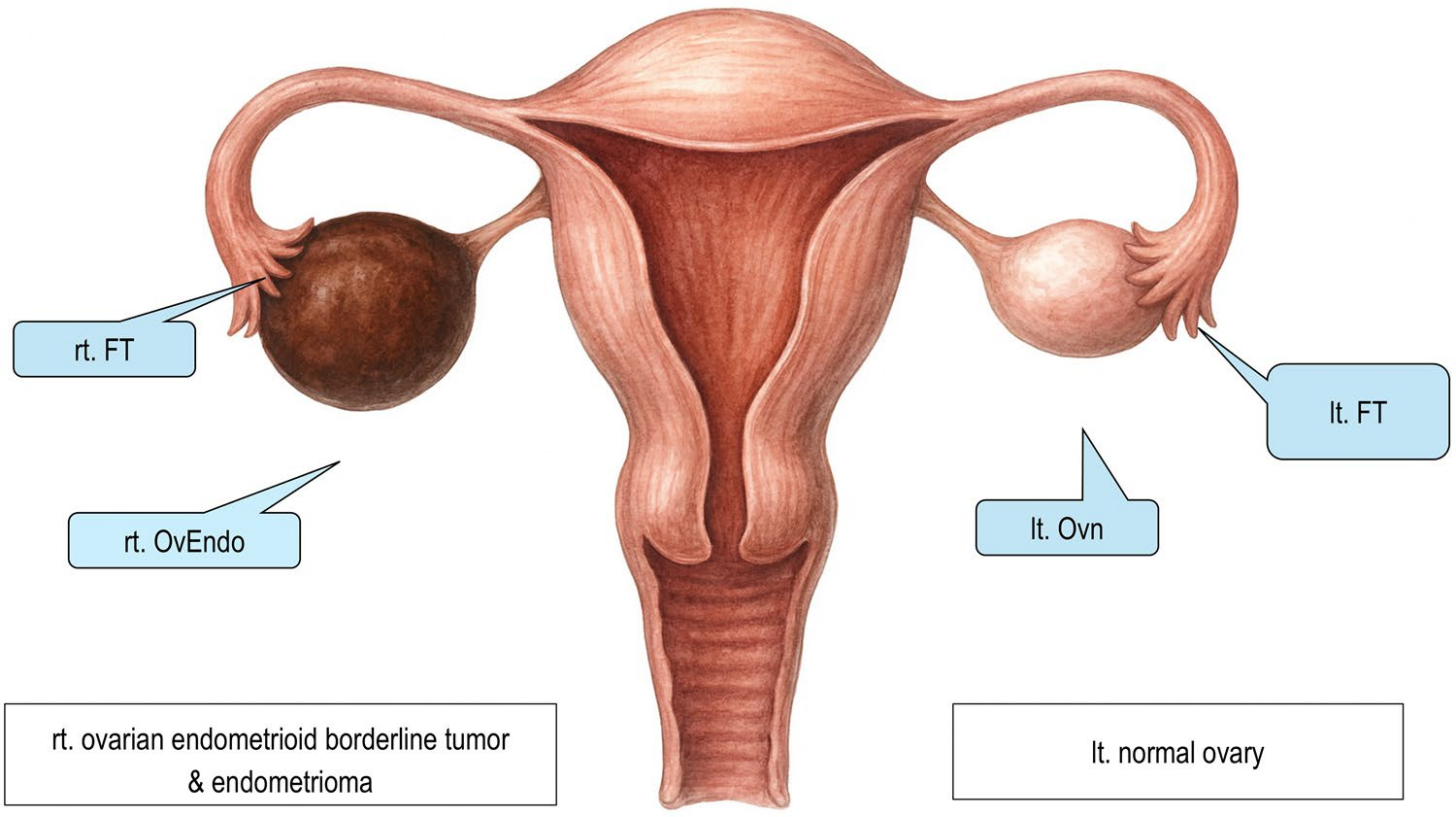
Schematic overview of sample collection and establishment of immortalized ovarian stromal cell lines

Figure 1 shows a schematic representation of the established cell lines. The cells were isolated and cultured from the inner wall of a right ovarian endometrioma (rt. OvEndo), the surface epithelium of the left ovary (lt. Ovn), bilateral fimbriae (right/left FT), and endometrial surface (Em).

The rt. Ov borderline tumor and Em cells did not proliferate because of insufficient cell quantity, which prevented the acquisition of an adequate cell volume necessary for SeV infection. The remaining cells were divided into Sendai virus-infected (immortalized cell lines) and non-infected (parental cells) groups and subsequently cultured.

Endometrioma lining, normal ovarian surface, and fimbrial tissues were collected from a single patient. After enzymatic digestion, primary cultures yielded mixed epithelial and stromal populations. During early passages, epithelial cells were lost, and fibroblast-like stromal cells were expanded and subjected to SeV-mediated immortalization. This workflow generated stable stromal cell lines suitable for long-term study of ovarian tumor–stroma interactions.

**Fig. 2:**
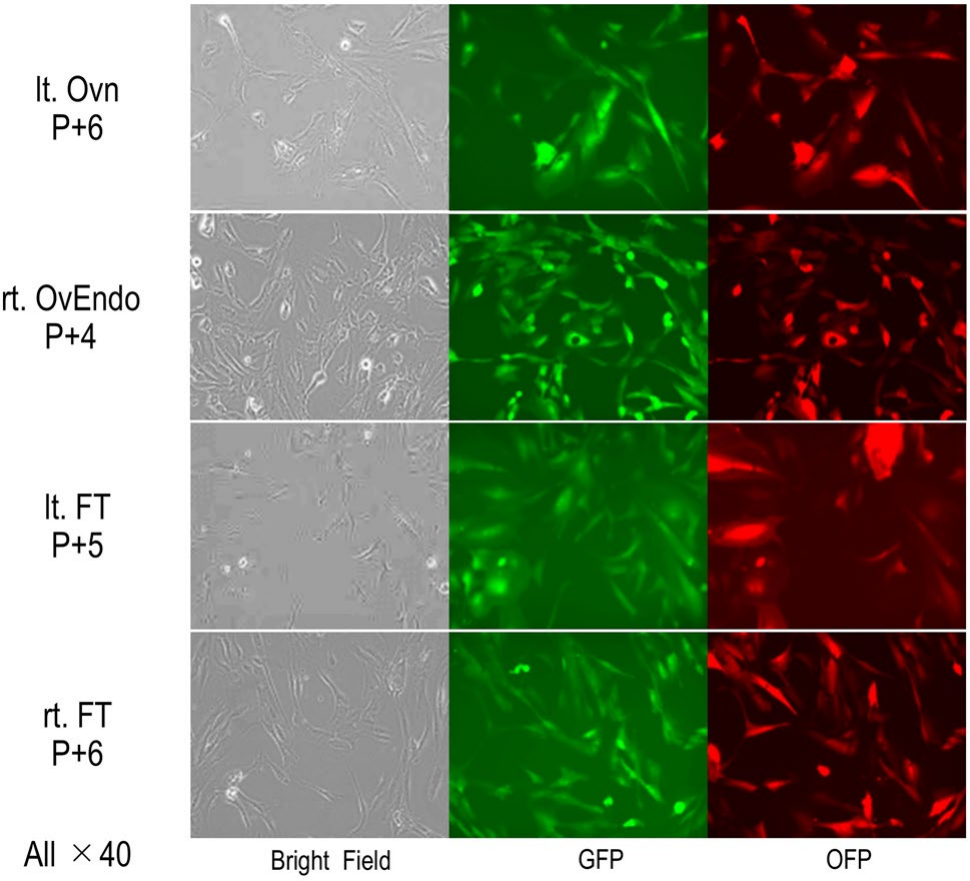
Confirmation of fluorescence staining using an all-in-one microscope

All cultured cells exhibited a spindle-, wedge-, or oval-shaped morphology. Green fluorescent protein and OFP fluorescence were detected in all cell lines (Fig. 2).

Fluorescence staining for green and orange fluorescent proteins was confirmed in all cell lines, including the left ovary, right ovarian endometrioma, left fimbriae, and right fimbriae.

### Establishment and characterization of immortalized ovarian stromal cells

**Fig. 3:**
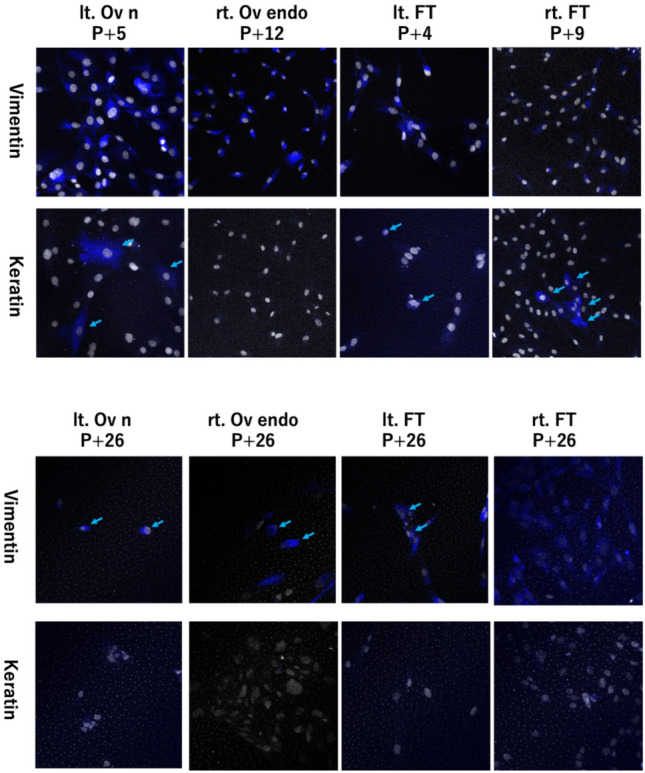
Immunocytochemistry (ICC)

Primary cultures from ovarian endometriomas, normal ovarian surface, and fallopian tubes yielded mixed epithelial–stromal populations. During early passages, epithelial cells were gradually lost, leaving predominantly fibroblast-like stromal cells. Using SeV-mediated transduction of TERT, BMI1, and SV40T, we successfully established immortalized stromal cell lines from these tissues. SeV-infected stromal cells exhibited spindle-shaped morphology, robust proliferation, and were maintained beyond 25 passages. In contrast, non-infected stromal cultures underwent senescence after ~ 5 passages. Immunocytochemistry confirmed the expression of stromal markers (Vimentin) and absence of epithelial markers (Keratin), verifying their stromal identity. These findings demonstrate that immortalized ovarian stromal cells can be generated and maintained long-term, providing stable models for ovarian tumor microenvironment research (Fig. 3).

Primary cultures from ovarian endometriomas, normal ovarian surface, and fallopian tubes yielded mixed epithelial–stromal populations. ICC confirmed expression of stromal markers (Vimentin) and epithelial markers (Keratin). The epithelial cells gradually disappeared, leaving predominantly fibroblast-like stromal cells. ICC confirmed Vimentin positivity and Keratin negativity, verifying stromal identity.

### Chromosomal stability and genomic alterations in stromal cells

**Fig. 4:**
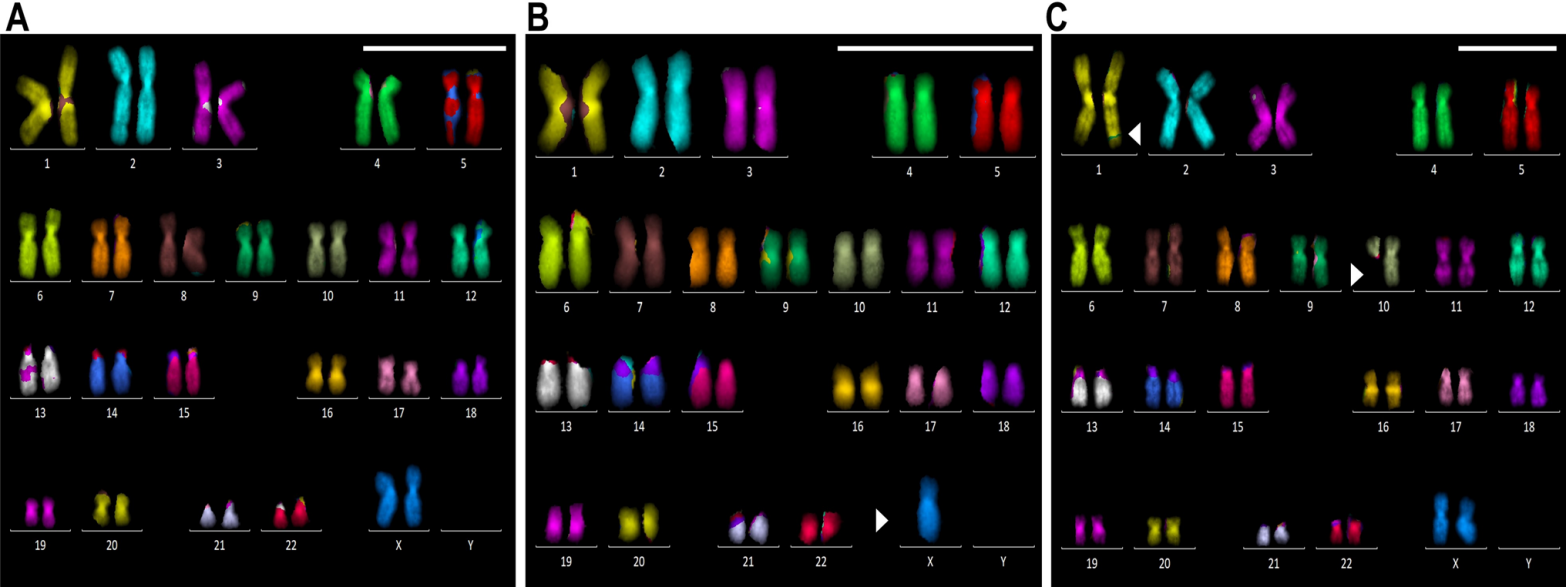
Confirmation of chromosomal structural abnormalities via mFISH. Karyotype notation does not conform to the ISCN. The white arrow indicates chromosomal rearrangement. Scale bar: 10 μm. A: rt. FT SeV⁺ P + 25, 46,XX, normal, B: rt. OvEndo SeV⁺ P + 25, 45,X, C: lt. Ovn SeV⁺ P + 25, 46,XX, t(8;9), Footnote: “SeV⁺” indicates immortalized cell lines established by Sendai virus-mediated transduction; “SeV⁻” indicates parental (non-infected primary) cells. “P + xx” denotes the passage number after infection (e.g., P + 25 = passage 25)

Cytogenetic analyses revealed numerical and structural chromosomal abnormalities in immortalized stromal cells, particularly those derived from endometriomas (rt. OvEndo) (Table 1). The frequency of abnormalities increased from 12% at early passages to > 50% after extended culture, with X-chromosome loss and 1;9 translocations identified (Fig. 4). By contrast, fallopian tube-derived stromal lines showed minimal abnormalities over long-term culture. These findings suggest that stromal cells in endometriomas harbor early genomic instability, consistent with their clinical potential to progress toward borderline or malignant tumors. Immortalized stromal lines, thus, provide an experimentally tractable system to study genomic instability in the ovarian stroma.

**Table 1:**
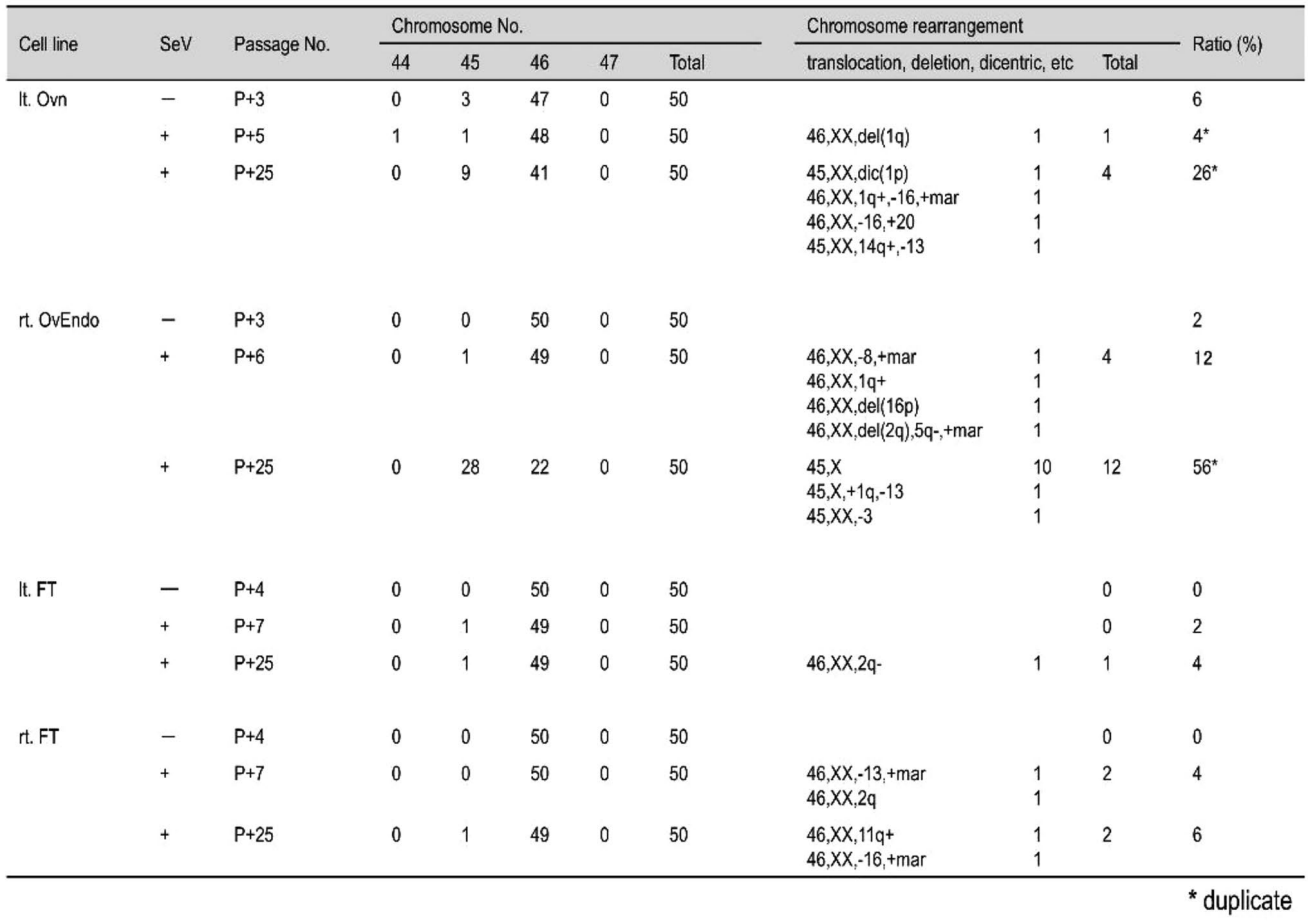
Numerical and structural chromosomal abnormalities

### Transcriptomic profiling of immortalized stromal cells

**Fig. 5:**
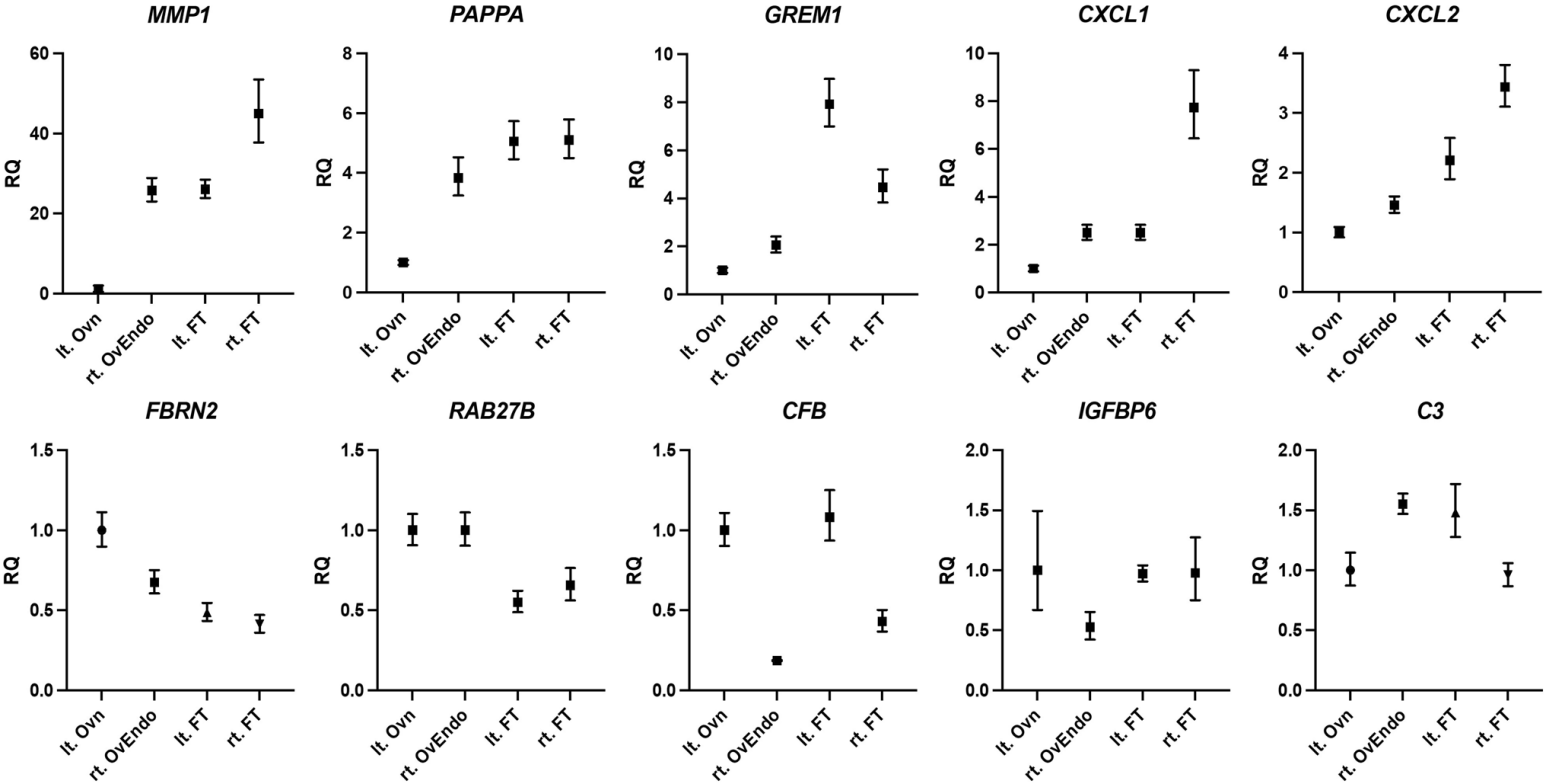
Gene expression comparison via RT-PCR. Both *MMP1* and *PAPPA* were highly expressed in rt. OvEndo than in lt. Ovn

The read lengths and numbers of assembled transcripts for each cell line are listed in Supplementary Table 1. The comparisons between the left ovary and right endometrioma, and between the left and right fallopian tubes, were performed to control for inter-individual variability, as all tissues were derived from the same patient. These intra-patient comparisons allowed us to distinguish disease-associated transcriptional changes from baseline inter-tissue differences within a single genetic background. The results of the cluster analysis for each sample are presented in Supplementary Fig. 1. Supplementary Table 2 lists the genes with significantly higher expression frequencies across the samples. RNA-seq and RT-PCR analyses revealed distinct transcriptional signatures between stromal lines derived from endometriomas and those from normal ovarian surface (Fig. 5). Endometrioma stromal cells showed significant upregulation of extracellular matrix and paracrine signaling genes, including MMP1, PAPPA, GREM1, and CXCL1, compared with normal ovary-derived stromal cells. Fallopian tube-derived stromal cells also displayed elevated levels of MMP1 and CXCL chemokines. These factors are implicated in matrix remodeling, angiogenesis, IGF signaling, and immune modulation, highlighting the tumor-promoting potential of ovarian stroma. The reproducibility of these profiles across immortalized lines demonstrates their value for functional studies of stromal contribution to ovarian cancer.

## Summary

Collectively, our results establish immortalized ovarian stromal cell lines as robust tools for cancer research. They overcome the limitations of short-lived primary cultures, retain stromal identity, and exhibit disease-relevant phenotypes such as genomic instability and dysregulated paracrine signaling. These properties underscore their utility in elucidating stromal mechanisms of ovarian carcinogenesis and in developing stromal-targeted interventions.

## Discussion

This study demonstrates the successful establishment of immortalized ovarian stromal cell lines using Sendai virus vectors carrying hTERT, BMI1, and SV40T. Our work highlights not only the technical feasibility of generating long-lived stromal models but also their biological and translational significance for ovarian cancer research. The immortalized stromal cells retained fibroblast-like morphology, expressed canonical stromal markers, and exhibited robust proliferation, providing a reliable experimental system for downstream studies.

Chromosomal analyses revealed that stromal cells derived from ovarian endometriomas displayed higher rates of numerical and structural abnormalities, including X-chromosome loss and chromosomal translocations, compared with stromal cells from normal ovary or fimbriae. This observation is consistent with clinical findings that endometriosis-associated stroma can undergo genomic alterations that predispose to borderline and malignant transformation. Thus, immortalized stromal lines provide an experimental platform to model genomic instability in endometriosis-related ovarian tumors.

Transcriptomic profiling further identified upregulation of MMP1, PAPPA, and CXCL chemokines in cyst-derived stromal cells, implicating extracellular matrix remodeling, IGF signaling, and inflammatory crosstalk as potential stromal drivers of ovarian carcinogenesis. These molecular changes are consistent with the known tumor-promoting functions of stromal fibroblasts, which contribute to invasion, angiogenesis, and therapeutic resistance. Recent single-cell RNA-seq analyses have identified distinct stromal subtypes in the human ovary, including fibroblastic, perivascular, and immune-modulatory stromal populations [8]. Based on marker expression (Vimentin + , Keratin–, MMP1 + , PAPPA +), our immortalized stromal lines most closely resemble fibroblastic stromal cells, suggesting that they model the fibroblast-like compartment responsible for ECM remodeling and paracrine signaling in ovarian carcinogenesis. The ability to capture and reproducibly study these gene expression patterns represents a major advantage of using immortalized stromal lines.

From a translational perspective, immortalized stromal cell lines are invaluable for modeling tumor–stroma interactions. They can be incorporated into advanced systems such as 3D co-cultures, patient-derived organoids, and tumor-on-chip models to recapitulate the ovarian tumor microenvironment more faithfully. By integrating these stromal lines, it will be possible to interrogate how stromal signaling supports cancer cell stemness, alters immune responses, and mediates drug resistance. In addition, stromal-specific pathways may serve as therapeutic targets for overcoming treatment resistance in ovarian cancer.

Our findings align with both basic and clinical research that highlight the significance of ovarian stromal biology. While immortalization approaches using hTERT or SV40T alone have been reported, these single-gene strategies are often limited by incomplete bypass of senescence or risk of transformation; in contrast, our SeV-based triple combination provides a more robust, non-integrating system for establishing stable stromal cell lines. On the basic side, immortalized epithelial cell models carrying BRCA1/2 mutations have successfully captured genetic instability in ovarian carcinogenesis [9]. Our stromal model complements these systems by providing a stable platform to study tumor–stroma interactions, thereby extending the mechanistic insights beyond the epithelial compartment. Clinically, elevated serum MMP-1 has been identified as an independent poor prognostic factor in ovarian cancer patients [10]. Since stromal fibroblasts are key producers of MMPs, our observation of stromal upregulation of MMP1 directly links the model to clinically relevant prognostic pathways.

By bridging epithelial immortalization studies and biomarker-based clinical evidence, our stromal lines demonstrate both biological and translational value. They offer a tractable system to investigate stromal mechanisms, such as MMP1-driven invasion and resistance, while aligning with pathways already validated as predictors of patient outcome.

This study has limitations. It represents a single case analysis, and further work is required to establish additional stromal lines to validate whether the observed genomic and transcriptomic changes are consistent across patients. Moreover, while SeV vectors avoid genomic integration, long-term culture itself may introduce alterations that must be monitored. Finally, epithelial cells were not successfully immortalized in this setting, underscoring the importance of optimizing co-culture and 3D microenvironmental conditions for future studies. A technical limitation of this study is that merged triple-channel ICC imaging (Vimentin/Keratin/nuclei) could not be acquired due to spectral interference between GFP/OFP reporters, the TO-PRO-3 nuclear stain, and commonly used immunofluorophores. As a result, stromal identity was confirmed using serial dual-channel staining rather than single-slide multiplex imaging.

In conclusion, the establishment of immortalized ovarian stromal cell lines overcomes a critical barrier in ovarian cancer research. These models preserve stromal identity, reveal disease-relevant genomic and transcriptomic alterations, and provide robust platforms for mechanistic studies and translational applications. Ultimately, immortalized stromal lines will be indispensable tools for dissecting the role of the ovarian stroma in carcinogenesis and for guiding the development of novel stromal-targeted therapies.

In addition, prior work demonstrates that CAF programs—such as MFAP5‑FAK–ERK signaling that modulates vascular permeability and drug delivery, and WNT5A‑driven niche support for ovarian cancer stemness—are tractable in stromal–epithelial models, reinforcing the value of stable stromal lines for mechanistic and translational studies [2, 11].

## Supplementary Information

Below is the link to the electronic supplementary material.

Supplementary file1 (DOCX 401 KB)

## Data Availability

The RNA-seq data generated in this study are not publicly available due to ethical and privacy considerations but are available from the corresponding author upon reasonable request. Other data supporting the findings of this study are included within the article and its supplementary materials.
