# Supplementary material for: Establishment of immortalized ovarian stromal cell lines using Sendai virus vectors: a platform for studying tumor–stroma interactions and carcinogenesis
Source: Hum Cell. 2025 Nov 26;39(1):15. doi: 10.1007/s13577-025-01324-6 (PMC12657542; doi:10.1007/s13577-025-01324-6)
Supplement: Supplementary file 1 — Supplementary file1 (DOCX 401 KB) [file 13577_2025_1324_MOESM1_ESM.docx]

**Supplementary Materials**

**Establishment of immortalized ovarian stromal cell lines using Sendai virus vectors: a platform for studying tumor–stroma interactions and carcinogenesis**

**Journal name:** Human Cell

Masayo Okawa^1^, Hiroaki Komatsu^1^, Yasuhiro Kazuki^2,3^, Kanako Kazuki^3^, Genki Hichiwa^2^, Kohei Hikino1, Yuki Iida1, Mayumi Sawada1, Hiroyuki Kugoh^4^, Shinya Sato^1^, Mitsuo Oshimura^3^, Tasuku Harada^5^, Fuminori Taniguchi^1^

1 Department of Obstetrics and Gynecology, Tottori University School of Medicine, Tottori, Japan

2 Department of Chromosome Biomedical Engineering, Integrated Medical Sciences, Graduate School of Medical Sciences, Tottori University

3 Chromosome Engineering Research Center, Tottori University

4 Division of Genome and Cellular Function, Department of Molecular and Cellular Biology,

5 Tottori University Hospital, Tottori, Japan

**Corresponding Author**

Hiroaki Komatsu, MD, PhD

Department of Obstetrics and Gynecology

Tottori University School of Medicine,

36-1 Nishicho, Yonago, Tottori 683-8504, Japan

Email: komatsu.h.med@tottori-u.ac.jp

Phone: +81-859-38-6647


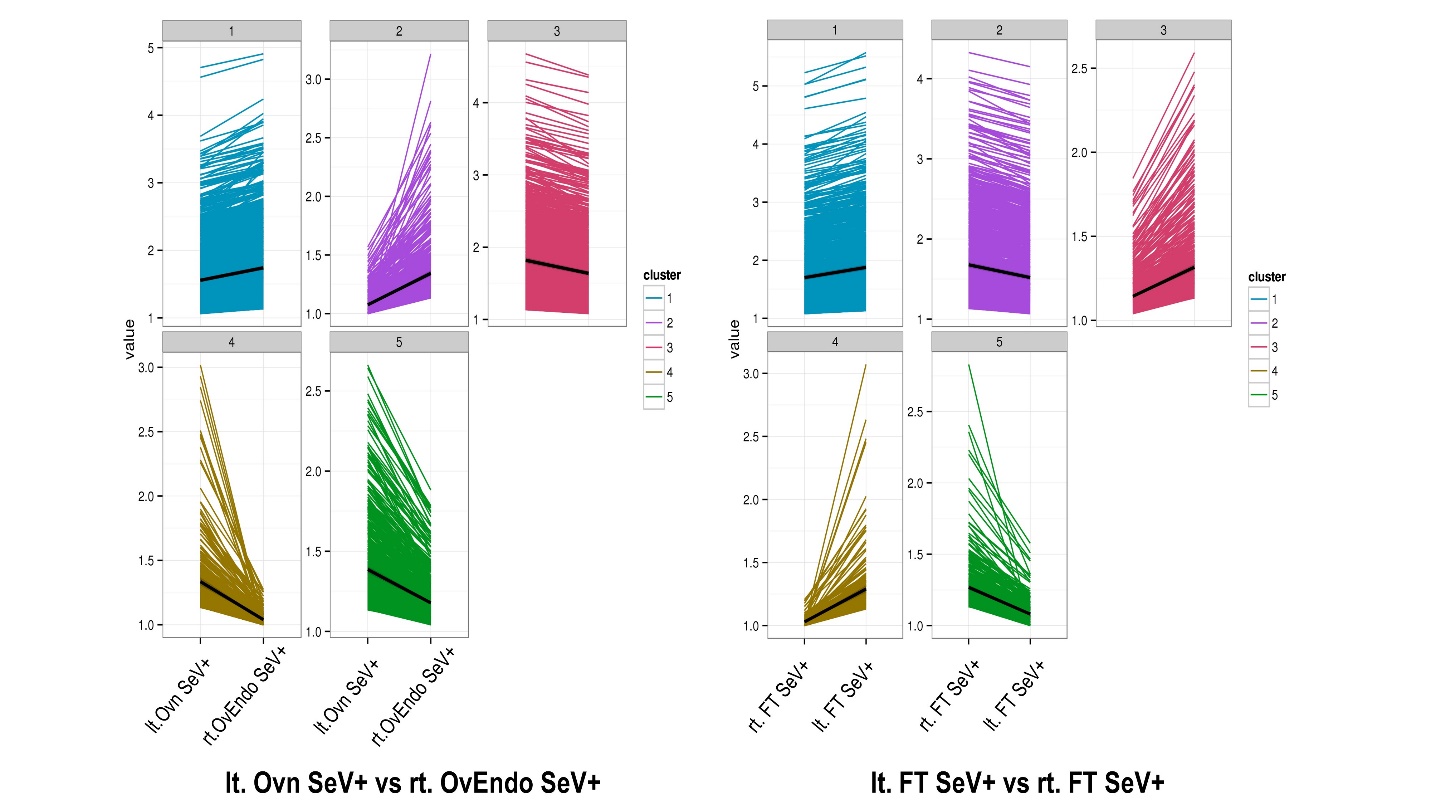


**Supplementary Figure 1** Clustering Analysis in Human Transcriptome Sequencing Analysis. The results of clustering analysis between the left ovary and right ovarian endometrial cyst and between the left and right fimbrae are shown. Each analysis classifies the samples into five clusters. FT, fimbrae; SeV, Sendai virus; Ovn, ovary; OvEndo, ovarian endometrial cysts.

Supplementary Table 1: Read Length and Number of Assembled Transcripts in Human Transcriptome Sequencing Analysis for Each Cell Line


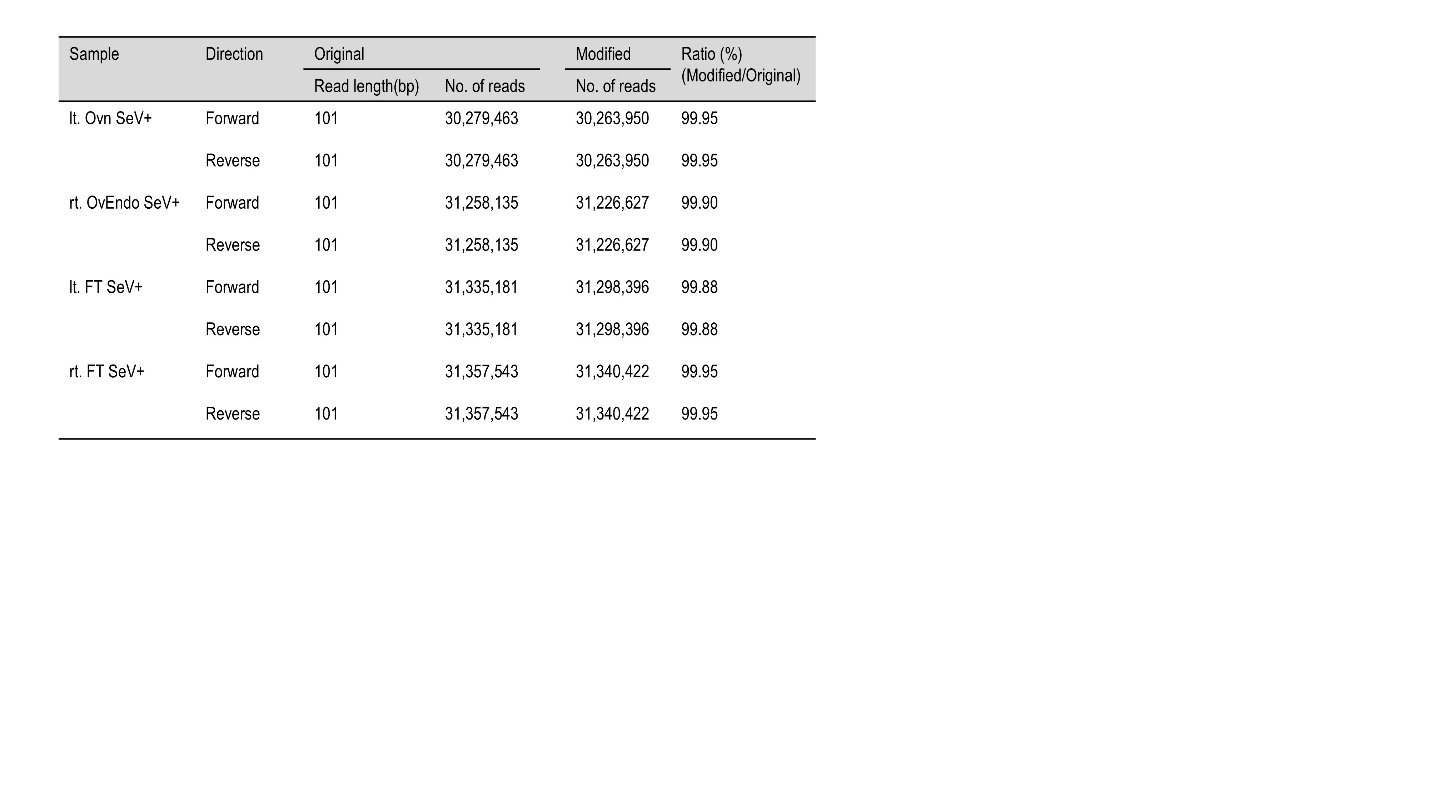


Supplementary Table 2: Human Transcriptome Sequencing Analysis


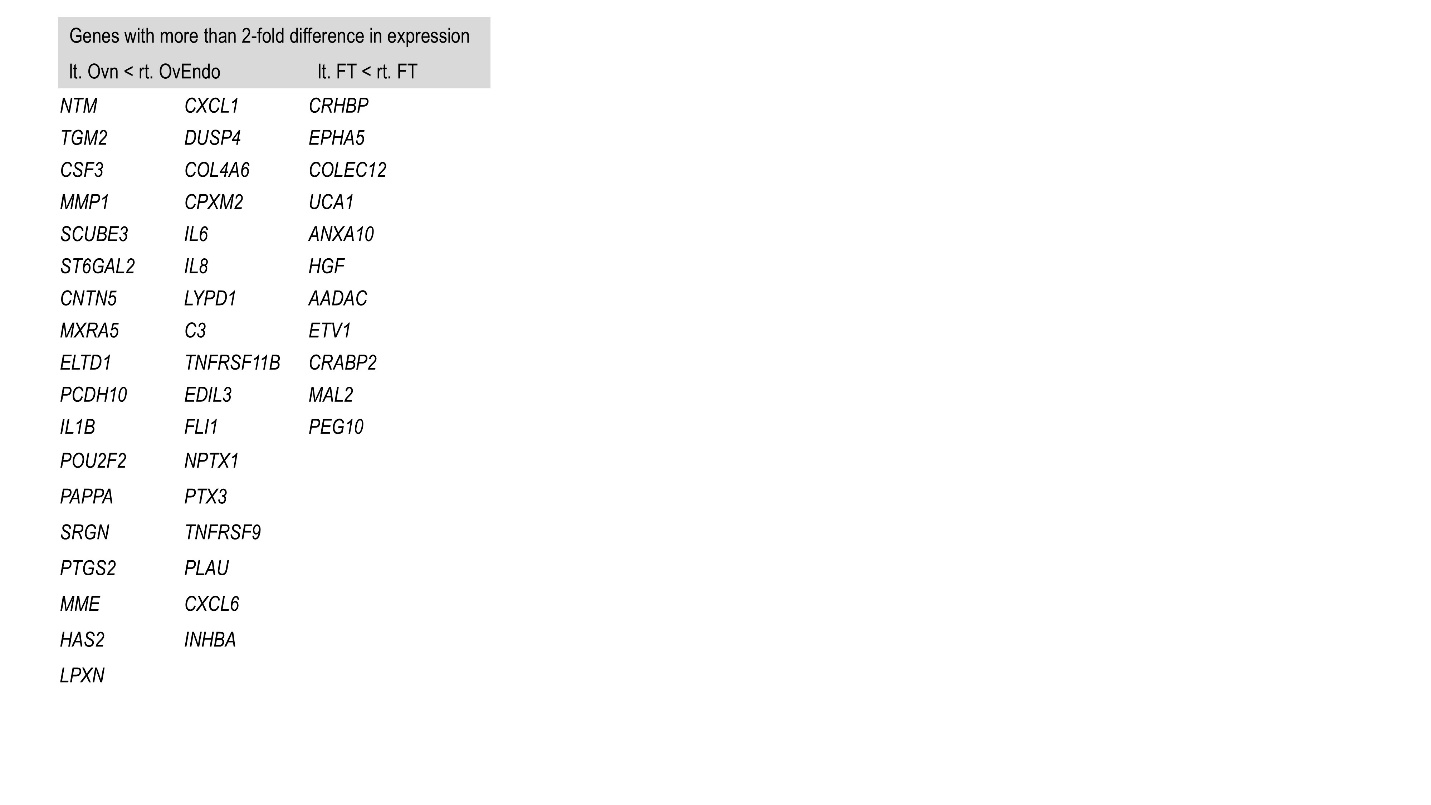


The genes *MMP1*, *PAPPA*, and *CXCL1* in the rt. OvEndo was expressed at levels more than twice as high as those of lt. Ovn
